# Tofacitinib effectiveness in Blau syndrome: a case series of Chinese paediatric patients

**DOI:** 10.1186/s12969-021-00634-x

**Published:** 2021-11-15

**Authors:** Song Zhang, Zhe Cai, Xiaolan Mo, Huasong Zeng

**Affiliations:** 1 Department of Allergy, Immunology and Rheumatology, Guangzhou Women and Children’s Medical Center, Guangzhou, 510120 Guangdong China; 2 Department of Pharmacy, Guangzhou Women and Children’s Medical Center, Guangzhou Medical University, Guangzhou, China

**Keywords:** JAK inhibitors, Tofacitinib, Blau syndrome, NOD2, Arthritis

## Abstract

**Objective:**

Blau syndrome (BS), a rare, autosomal-dominant autoinflammatory syndrome, is characterized by a clinical triad of granulomatous recurrent uveitis, dermatitis, and symmetric arthritis and associated with mutations of the nucleotide-binding oligomerization domain containing 2 (NOD2) gene. Aim of this study was to assess the efficacy of tofacitinib in Chinese paediatric patients with BS.

**Methods:**

Tofacitinib was regularly administered to three BS patients (Patient 1, Patient 2, and Patient 3) at different dosages: 1.7 mg/day (0.11 mg/kg), 2.5 mg/day (0.12 mg/kg), and 2.5 mg/day (0.33 mg/kg). The clinical manifestations of the patients, magnetic resonance imaging results, serological diagnoses, therapeutic measures and outcomes of treatments are described in this report.

**Results:**

The clinical characteristics and serological diagnoses of all BS patients were greatly improved after the administration of tofacitinib treatment. All patients reached clinical remission of polyarthritis and improvements in the erythrocyte sedimentation rate (ESR) and levels of C-reactive protein (CRP) and inflammatory cytokines.

**Conclusion:**

Tofacitinib, a Janus kinase (JAK) inhibitor, is a promising agent for BS patients who have unsatisfactory responses to corticosteroids, traditional disease-modifying antirheumatic drugs, and biological agents.

## Background

Blau syndrome (BS) and early-onset sarcoidosis (EOS) are rare monogenic autoinflammatory diseases resulting from mutations of the nucleotide-binding oligomerization domain containing 2 (NOD2)/caspase recruitment domain family member 15 (CARD15) gene on chromosome 16 [1, 2]. BS is characterized by the syndrome of arthritis, uveitis, skin rash and granulomatous inflammation [3, 4]. The NOD2 gene is located on chromosome 16q12 and is highly expressed in antigen-presenting cells, such as monocytes and macrophages, and in intestinal Paneth cells [5]. CARD15 is expressed as a cytoplasmic monomer and constituted with 1040 amino acids. CARD15 has two isoforms, a full-length product (isoform 1) and isoform 2, which share an alternative initiation site corresponding to amino acid position 28. Both isoforms can activation of nuclear factor-κB (NF-κB) is a key step in the upregulation of many genes involved in inflammatory cascades [6]. It is critical to control the eye and joint involvements to improve the prognosis of BS. To date, treatments consist of non-steroid anti-inflammatory drugs, corticosteroids, methotrexate and azathioprine. Recently, biologic agents such as tocilizumab, interleukin (IL)-1 blockers and tumour necrosis factor (TNF)-α inhibitors have demonstrated effective in some cases [7–9]. In this study, we collected clinical data and laboratory studies from patients with BS. A missense heterozygous mutation in the NOD2 gene was revealed in each patient and their pedigrees. Furthermore, Janus kinase (JAK)-signal transducer and activator of transcription (JAK-STAT) are constitutively activated in sarcoidosis [10]. BS and EOS are paediatric granulomatous autoinflammatory syndromes that belong to a group of monogenic autoinflammatory syndromes [11–13]. JAK-STAT activation has been demonstrated to lead to an increase of levels of cytokines, such as interferon-γ (IFN-γ) and IL-6, that are produced by T cells and macrophages and play important roles in the pathogenesis of BS/EOS. Therefore, It appeared reasonable that inhibiting the JAK-STAT pathway could be an effective treatment strategy for BS/EOS. The treatment of BS/EOS in child patients is challenging. It also appeared reasonable that biologic treatment for BS may have diverse results due to varying genotypes and phenotypes of BS [10, 11].

In this study, we describe three Chinese patients at the Guangzhou Women and Children Medical Center. All mutations were located on exon 4 of the NOD2 gene; Patient 1 and Patient 2 (R334Q) had recurrent BS mutations [1]; however, a novel, unreported mutation in NOD2, p.M513K, but not p.M513T or p.M513R [2, 14], was found in Patient 3 by whole-exome sequencing. All of the patients were followed-up for more than half a year. Complete medical records and detailed clinical parameters were collected for further analysis. We assessed the therapeutic effect of treatment by measuring the white blood cell count (WBC); C-reactive protein (CRP) level; erythrocyte sedimentation rate (ESR); and the levels of TNF-α, IL-1β, and IL-6. This research was approved by the Ethics Committee of Guangzhou Women and Children Medical Center and performed according to the Declaration of Helsinki. Informed consent was obtained from all participants. Whole-exome sequencing was performed at the Center for Genetic Testing, Makino Beijing Gene Technology Co. Ltd., China.

## Case presentation

**Fig. 1:**
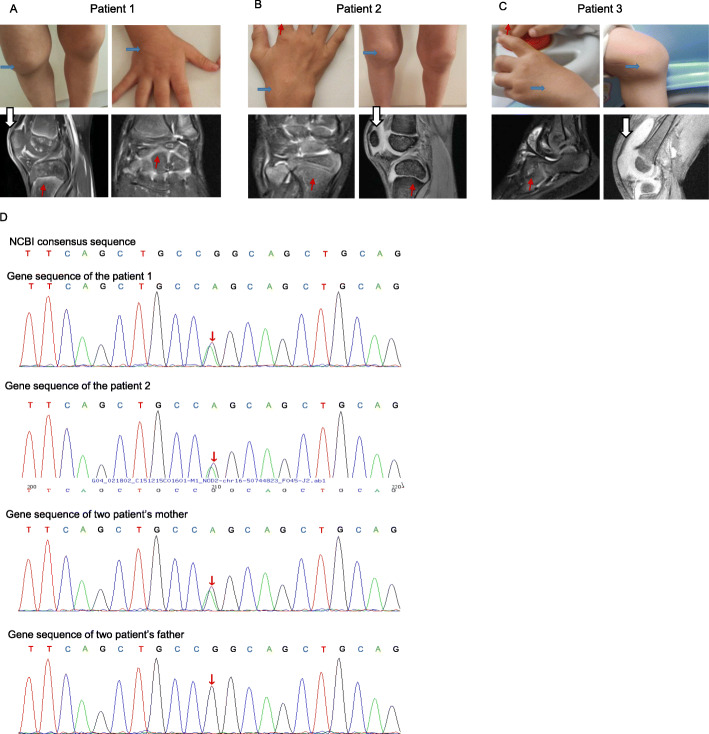
Clinical features of the patients. **A** Patient 1 had multiple swollen joints ()including joints in both hands, knees, ankles and feet. Nuclear magnetic resonance (NMR) imaging of the wrist, knee and ankle joints of Patient 1 were obtained before tofacitinib treatment in December 2015. Synovitis (), joint effusion and inflammatory oedema of the bone () and periarticular soft tissue were observed in the ankle and knee joints. **B** Features of paediatric granulomatous arthritis of Patient 2. Symmetric arthritis affecting the wrists, metacarpophalangeal and proximal interphalangeal joints, and knee in June 2018. NMR imaging of the knee and wrist joints of Patient 2 was carried out before tofacitinib treatment. Synovitis, joint effusion, and inflammatory oedema of the bone and periarticular soft tissue were observed in the wrist and knee joints. **C** Features of paediatric granulomatous arthritis of Patient 3. Symmetric arthritis affecting the wrists, ankle and knee. Nuclear magnetic resonance imaging (NMR) of the knee and ankle of Patient 3 in July 2019. Synovitis, joint effusion, inflammatory oedema of bone and periarticular soft tissue were observed in the wrist and knee joints. **D** Identification of the NOD2 R334Q (1001G>A) mutation in Patient 1, Patient 2 and their mother

**Fig. 2:**
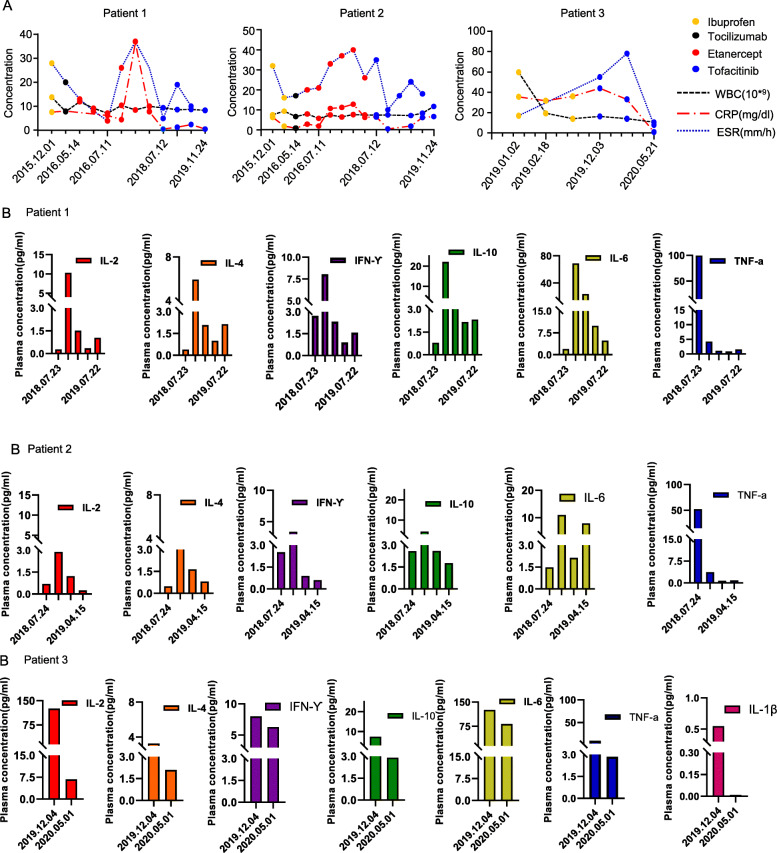
The laboratory results for the 3 patients. **A** Laboratory results for the patients, including WBC, CRP level and ESR. **B** Plasma levels of the proinflammatory cytokines IL-2, IL-4, IL-6, IL-10, TNF-α and IFN-γ in Patient 1 and Patient 2. Plasma levels of the proinflammatory cytokines IL-2, IL-4, IL-6, IL-10, TNF-α, IFN-γ and IL-1ß in Patient 3

Case report, Patient 1: The patient, A 16 month-old male child was brought to our clinic for arthritis. The onset of the arthritis was at age 8 months and included the knee, ankle, hand, foot and fingers. The patient presented with tender and swollen joints (knee, ankle, elbow, wrist, metacarpophalangeal joints, toe), and knee deformities caused by arthralgia. No fever, rash, or uveitis had been documented (Table 1 and Fig. 1A). Nuclear magnetic resonance (NMR) imaging showed synovitis, joint effusion and inflammatory oedema of the bone and periarticular soft tissue (Fig. 1A). Initially, at an age 16 months, he was diagnosed with systemic juvenile idiopathic arthritis. He was treated with ibuprofen, methotrexate (MTX), and prednisone; yet the symptoms persisted. Lab tests were again abnormal including WBC, ESR and CRP (Fig. 2A). Moreover, At 18 months of age, a genetic analysis revealed heterozygous missense mutation (R334Q) in the NOD2 region of the NOD2/CARD15 gene (Table 1 and Fig. 1D), He was diagnosed to have Blau syndrome at that point. He was begun on tocilizumab for 1 month without improvement. He then received a 2 year course of methotrexate and etanercept but still continued to have swollen and painful joints. His ESR varied between 4 and 26 mm/hour, his WBC 8.4–10.4 × 109 (ninth power)/liter, and his CRP level from 0.5–7.8 mg/dL (Fig. 2A). Plasma cytokines remained elevated despite this biologic and DMARD treatment (Fig. 2B). Etanercept treatment was stopped, and tofacitinib was orally administered starting on July 2018 with a final dosage of 1.7 mg/day. After 1 year of tofacitinib, his arthritis was in clinical remission and his lab tests were in the normal range.

**Table 1:** Overview of participants and response rates per school grade over 23 weeks, including retrospective data

Patients	1	2	3	References
Gender	Male	Male	Male	
Ethnicity	Han	Han	Han	
Age at diagnosis (years)	1.5	6.8	1.7	
Age at onset (years)	0.8	0.6	0.5	
Family history	+	+	-	
Clinical features				
Joint	+	+	+	
Skin	-	-	+	
Eye	-	-	-	
Fever	-	-	-	
NOD2 variants	R334Q	R334Q	p.M513K	
Laboratory findings				
WBC (10^9^/L)	13.8	7.5	59.6	5-12
CRP (mg/L)	7.6	6.2	35.5	≤8.2
ESR (mm/h)	28	32	17	0-15
IL-2 (pg/ml)	0.28	0.69	125.74	0-5.03
IL-4 (pg/ml)	0.39	0.69	3.26	0-4.62
IL-6 (pg/ml)	1.96	0.69	125.49	0-8.88
IL-10 (pg/ml)	0.79	0.69	7.42	0-8.14
TNF-α (pg/ml)	99.47	0.69	9.52	0-5.35
IFN-γ (pg/ml)	2.73	0.69	7.99	0-6.56
IL-1ß (pg/ml)			0.55	0-3.12

Case report, Patient 2: The brother of patient 1 presented to the clinic at age 6 years and 10 months. He had developed chronic arthritis at age 6 months involving his ankles, knees, elbows, toes, and metacarpophalangeal joints. Like his brother, he had no history of rash, fevers, or uveitis (Table 1). He had been diagnosed to have systemic JIA at another hospital at age 5 years. At that time, he was started on ibuprofen and etanercept but did not improve. When he was seen in this hospital at almost 7 years of age, radiographs of his ankles, knee, and fingers showed joint damage (Fig. 1B). His ESR ranged from 16 to 32 mm/hour, WBC 7.5–9.3 × 109 (ninth power)/liter and CRP levels of 1.8–6.2 mg/dL (Fig. 2B). Genetic analysis appeared warranted and revealed a heterozygous missence mutation (R334D) in the NOD-2 region of the CARD15/NOD-2 gene (Table 1, Fig. 1D) (8). He was then diagnosed to have Blau syndrome. After the diagnosis of Blau syndrome, he was initially treated with methotrexate and tocilizumab with little response. He was then switched to subcutaneous methotrexate and etanercept. He improved for 1–2 months but then relapsed with abnormal labs (ESR 10–40 mm/hour, WBC 5.7–11.7 × 109 (ninth power) and CRP of 0.4–12.8 mg/dL). At the age of 9.3-years-old, after stopping etanercept, oral administration of tofacitinib was started with a final dosage 2.5 mg/day in July 2018. The tofacitinib therapy appeared to relieve his joint pain and swelling and was associated with a major decrease in his ESR and cytokine levels (Fig. 2A, B).

Case report, Patient-3. A 5 month-old infant developed arthritis in his ankles and wrists and a rash. No fever or uveitis problems were obvious (Table 1, Fig. 1C). His ESR was 17–55 mm/hour, his WBC 12.9–68.6 × 109 ninth power/L, and CRP 2.05–47.7 mg/dL (Fig. 2A). The levels of cytokines (IL-1B, IL-2, IL-6, IFN-γ, and TNF-α) were increased as well (Fig. 2B). NMR imaging of his knees and ankles in 2019 revealed synovitis, joint effusions, and inflammatory oedema of the bone and periarticular soft tissue (Fig. 1C). At that time, he was diagnosed with systemic onset juvenile idiopathic arthritis and begun on ibuprofen and methotrexate; however, no improvement followed. Genetic analysis at 21 months of age revealed a novel heterozygous missense mutation (p.M513K) in the NOD2 region of the NOD2/CARD15 gene (Table 1). Sequence data showed that the parents did not carry this mutation. He was diagnosed with BS based on the mutation and a compatible clinical scenario. He was started on tofacitinib 2.5 mg per day. The treatment appeared to improve his joint pain and swelling greatly over the next 4 months and his ESR, WBC, and CRP also decreased during that 4 month period (Fig. 2B).

## Discussion

**Fig. 3:**
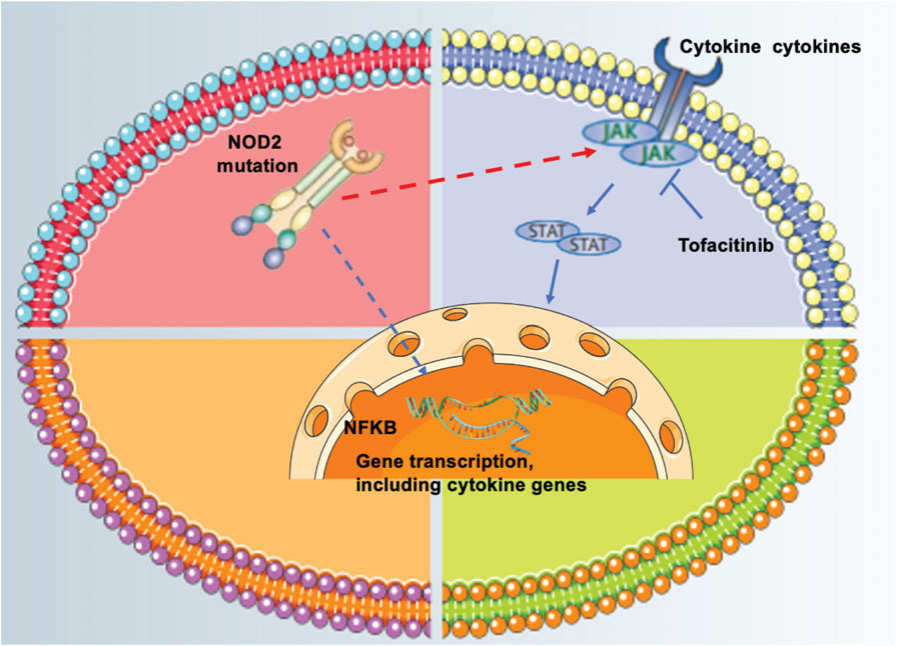
Possible model of the activation of JAK and secretion of proinflammatory chemokines induced by the NOD2 mutation and their interference by tofacitinib

Blau syndrome is a rare systemic granulomatous disorder associated with NOD-2 mutations. NOD-2 mutations have been associated with other chronic inflammatory illnesses such as Crohn’s disease and EOS. NOD-2 is needed for induction of NFkB. It’s leucine-rich repeats, though, are apparently dispensable [5, 15–17]. The activation of NF-κB induced by NOD2 may rely on induced proximity of downstream effectors, including the RIP-like interacting CLARP kinase (RICK) and inhibitor of NF-κB (IκB)-kinase (IKK) complex, and promote caspase activation, which leads to secretion of the proinflammatory cytokines IL-1, IL-6, and TNF-α [18, 19]. BS and EOS are caused by mutations in the NOD2 gene, which encodes the cytosolic NOD2 protein; this protein is a pivotal molecule in the regulation in immunity that is primarily expressed in antigen-presenting cells [11, 17, 20–23]. Studies have shown that JAK-STAT signalling is constitutively activated in sarcoidosis [10, 24–26], In sarcoidosis, dysregulated signalling of cytokines, such as IFN-γ and IL-6, which help to develop granuloma formation, occurs via the JAK-STAT pathway (Fig. 3) [10]. A JAK inhibitor has produced rapid clinical improvements in RA patients who had previously failed other disease-modifying antirheumatic drug therapies or TNF antagonists [10, 27]. JAK inhibitors disrupt γc-chain cytokine signalling in CD4^+^ Th cells and block IL-6 signalling, which rapidly suppresses collagen-induced arthritis, the expression of inflammatory cytokines, and STAT1-dependent gene expression and interferes with the differentiation of Th1 and Th2 cells and the generation of inflammatory cytokines, such as IL-1β, IL-6 and IL-23, by Th17 cells [28].

The mRNA levels of proinflammatory cytokines (such as IL-6, TNF-α, and IL-1β) have been reported to be increased in BS [29]. Accordingly, biological agents such as IL-1 and IL-6 inhibitors and TNF inhibitors have been used to treat BS [6]. Anti-TNF agents, such as infliximab, etanercept, adalimumab, and thalidomide, have shown promising results in several studies [6, 7, 9, 29] and are currently the most commonly used biologic therapies for BS in China. In three patients, we found that a single dose of the JAK inhibitor tofacitinib suppressed TNF and IL-6 production along with the production of other inflammatory cytokines. Substantial improvements in clinical symptoms and laboratory parameters in the tofacitinib-treated patients were observed. The symptoms of Patient 1 and Patient 2, who were treated with the TNF-α inhibitor and tocilizumab, have not yet improved. Replacement TNF-α inhibitor treatment resulted in relapse within the first year, and the joints began to swell again. It is speculated that TNF-α may not be the only cytokine that plays an important role in the pathogenesis of BS. In these 3 patients, TNF-alpha inhibitor therapy failed. This failure may suggest that TNF-alpha may not be the most important or the only important cytokine for BS. The reports and our results suggest that it is best to try a non-TNF inhibitor such as abatacept or tocilizumab if the initial TNF-alpha biologic does not help [30–32]. This report suggests that tofacitimib therapy may be a good option. There may be several reasons for this usefulness of tofacitimib treatment in BS but certainly one explanation might be that this drug inhibits not just TNF-alpha but appears to inhibit IL-1, IL-6, and IFN-gamma cytokines. Our 3 patients had no side effects in their short treatments of 1 year.

## Conclusions

This case series reports the successful treatment of Blau syndrome in 3 young children using tofacitinib. Arthritis, uveitis, and rash improved on this drug after treatment failures of methotrexate, tocilizumab, and etanercept. No side effects were seen. This drug merits a multicenter study for Blau’s syndrome.

## Data Availability

Not applicable.

## Abbreviations

BS: Blau syndrome
CARD15: caspase recruitment domain family member 15
CRP: C-reactive protein
EOS: early-onset sarcoidosis
ESR: erythrocyte sedimentation rate
NF-ne: nuclear factor-κB
NOD2: nucleotide-binding oligomerization domain containing 2
RICK: RIP-like interacting CLARP kinase
TNF: tumour necrosis factor
WBC: white blood cell count
IKK: inhibitor of NF-κB (IκB)-kinase
IL: interleukin
IFN-γ: interferon-γ
JAK: Janus kinase
JAK-STAT: Janus kinase-signal transducer and activator of transcription
